# Genetic and clinical evidence implicates a potential Parasutterella–sphingomyelin pathway in diabetic nephropathy

**DOI:** 10.3389/fmicb.2026.1848678

**Published:** 2026-07-16

**Authors:** Chunling Huang, Jing Zhou, Hongtao Zhang, Yue Gu, Fengmin Shao

**Affiliations:** 1 Department of Nephrology, Zhengzhou University People's Hospital, Henan Provincial People’s Hospital, Zhengzhou, China; 2 Henan Provincial Clinical Research Center for Kidney Disease of Zhengzhou University People’s Hospital, Henan Key Laboratory of Kidney Disease and Immunology of Zhengzhou University People’s Hospital, Henan Provincial People’s Hospital, Zhengzhou, China; 3 Blood Purification Center, Zhengzhou University People's Hospital, Zhengzhou, China

**Keywords:** diabetic nephropathy, gut microbiota, mediation analysis, Mendelian randomization, plasma metabolites, sphingomyelin

## Abstract

**Background:**

The gut microbiota is closely associated with the onset and progression of diabetic nephropathy. We aimed to define metabolite mediators in this pathway and to validate their relevance in patient samples and podocyte models.

**Methods:**

We performed Mendelian randomization and colocalization analyses on 889 gut microbiota features and 1,400 metabolic biomarkers for diabetic nephropathy risk, estimated glomerular filtration rate, and urine albumin-to-creatinine ratio. Mediation analysis evaluated potential indirect microbiota effects through metabolites, and a multi-step genetic locus strategy was used to prioritize candidate variants. We then conducted plasma metabolomics in healthy controls, patients with diabetes mellitus without DN, and patients with biopsy-confirmed DN, assessed correlations with eGFR and creatinine, quantified total sphingomyelin, and performed sphingomyelin intervention in human podocytes under high-glucose conditions.

**Results:**

After excluding reverse causality, 25 species, 22 higher-level taxa, and 12 microbial metabolic pathways were associated with at least one diabetic nephropathy phenotype. Mediation analysis prioritized three candidate microbiota-metabolite-DN pathways, including a potential genus Parasutterella-sphingomyelin (d18:1/24:1, d18:2/24:0)-DN pathway. Colocalization analysis provided exploratory support for a possible shared genetic signal in this candidate pathway. Seven candidate metabolites were detected in patient plasma, and exploratory group comparisons showed that SM (d18:1/16:0) differed among groups. SM (d18:1/16:0) was negatively correlated with eGFR and positively correlated with creatinine, and total sphingomyelin was markedly elevated and correlated with renal dysfunction. In podocytes, sphingomyelin treatment under high-glucose conditions aggravated injury-related, fibrotic/extracellular matrix-related, and inflammatory-related changes, supporting a potential functional role of sphingomyelin dysregulation under DN-related injury conditions.

**Conclusion:**

This integrative analysis provides genetic evidence supporting potential links among gut microbiota, plasma metabolites, and DN. The results prioritize a potential Parasutterella-sphingomyelin-DN pathway, while exploratory clinical and experimental data support sphingomyelin dysregulation in DN, particularly under established renal injury or high-glucose conditions.

## Introduction

Diabetic nephropathy (DN) is a microvascular complication of diabetes mellitus (DM) that significantly impacts patients’ survival and prognosis. According to the International Diabetes Federation report, an estimated 536.6 million adults aged 20–79 years worldwide have diabetes, with approximately 40% of type 2 DM patients progressing to DN. The report also noted that the number of patients with chronic kidney disease caused by DM increased from 1.4 million in 1990 to 2.4 million in 2017 (Sun et al., 2022). As one of the severe complications of DM, DN is imposing an increasingly heavy burden on social medical security systems and public finances due to its high incidence and complex diagnostic and therapeutic requirements.

The gut microbiota (GM) is an important regulator of host metabolic and immune homeostasis. It contributes to intestinal barrier function, immune regulation, and nutrient metabolism through the production of bioactive metabolites. With advances in 16S rRNA gene sequencing and metagenomic technologies, interest in the role of GM in metabolic and renal diseases has grown rapidly. Several studies have reported that patients with chronic kidney disease show marked alterations in GM composition, including reduced microbial diversity and abnormal accumulation of microbial-derived metabolites (Anders et al., 2013; Meijers et al., 2019). Recent observational studies, animal experiments, and Mendelian randomization (MR) analyses have suggested a possible link between GM and DN (Qin et al., 2012; Linh et al., 2022; Chen P. P. et al., 2023; Wu et al., 2025). However, the available evidence remains inconsistent, and it is still unclear whether GM has a causal role in the development of DN.

Metabolic dysregulation is a central feature of diabetes and plays a critical role in the development and progression of DN (Fang et al., 2023). Abnormal glucose and lipid metabolism, insulin resistance, obesity, and chronic low-grade inflammation have all been linked to renal injury and increased risk of DN. Notably, metabolites derived from or modulated by the GM are implicated in glucose and lipid metabolism disorders, which are central to DN progression (Yang et al., 2021). These metabolites can influence key metabolic pathways, inflammatory responses, and endothelial function (Pongrac Barlovic et al., 2020; Gordin et al., 2019; Qi et al., 2017). Increasing evidence suggests that altered circulating levels of microbial-related metabolites are associated with metabolic disorders and kidney disease. This raises the hypothesis that GM may influence DN risk through modulating circulating metabolite levels. However, despite observational associations, causal evidence linking GM, specific metabolites, and DN remains scarce, and the underlying pathways are not defined.

Therefore, we conducted a two-sample MR study to estimate the potential causal effects of GM on DN-related phenotypes and to examine whether circulating plasma metabolites may mediate these effects, supported by colocalization analysis to assess possible shared genetic signals.

## Materials and methods

### Study protocol

**Figure 1:**
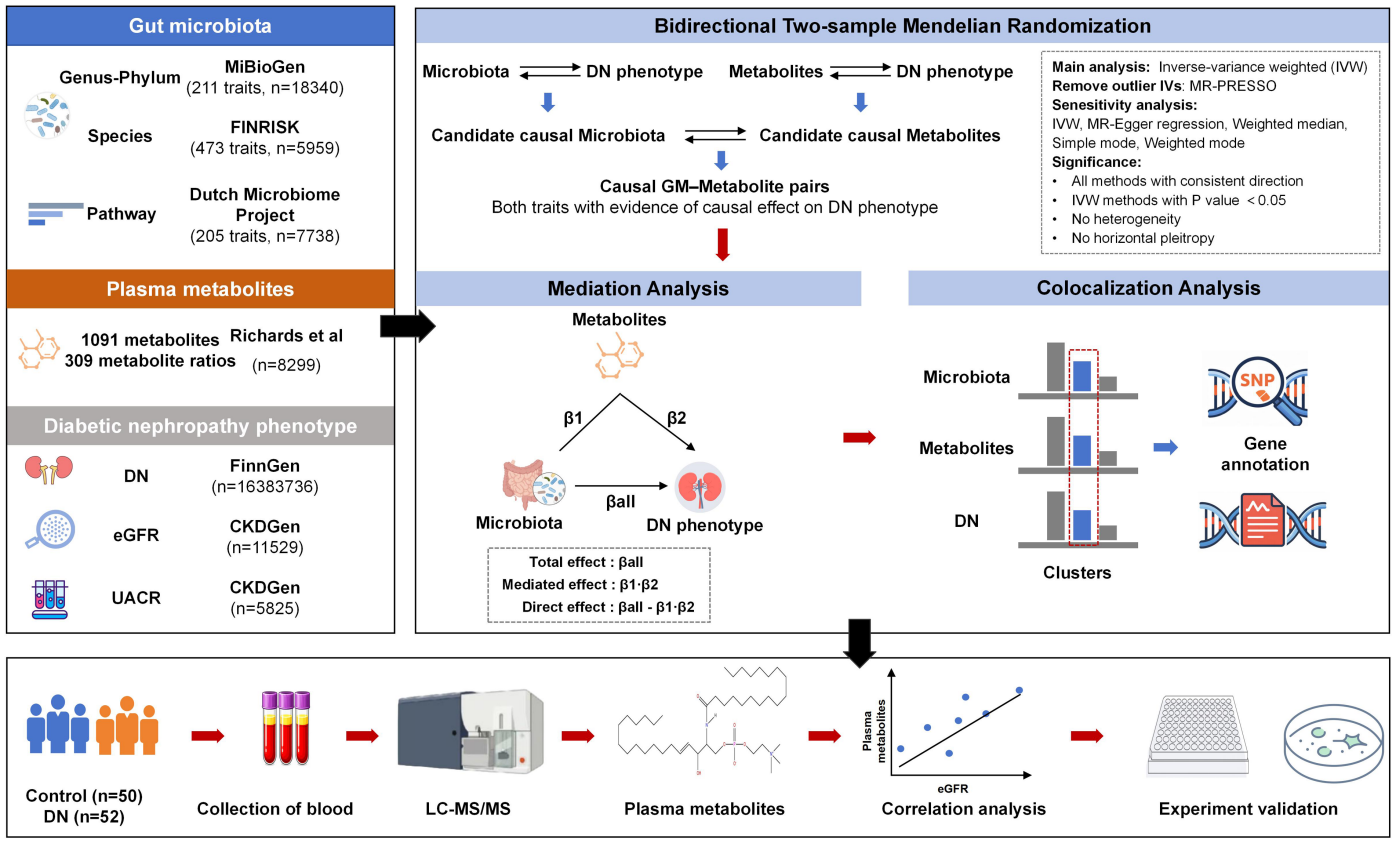
Schematic overview of the study design.

This study was designed as a two-sample MR analysis to investigate the potential causal effects of GM on DN-related phenotypes. Summary-level genome-wide association study (GWAS) data were used for GM, circulating plasma metabolites, and DN phenotype. The schematic of the study design was shown in Figure 1. First, we performed bidirectional two-sample MR to identify gut microbiota and metabolites with MR-supported potential causal associations with DN, estimated glomerular filtration rate (eGFR), or urinary albumin-to-creatinine ratio (UACR). Next, we conducted bidirectional MR between the candidate microbiota and metabolites. Pairs showing MR evidence of potential causal effects and associations with DN-related traits were selected for mediation analysis. Finally, we estimated potential indirect causal effects of microbiota on DN through metabolites. Colocalization analysis was used to provide exploratory evidence for possible shared genetic signals among microbiota, metabolites, and DN. All analyses followed the Strobe-MR guidelines.

### Data sources

Genetic instruments for GM were obtained from three large-scale GWAS datasets. These included the MiBioGen consortium GWAS (Kurilshikov et al., 2021) of 211 bacterial taxa (*n* = 18,340), the FINRISK 2002 gut microbiome GWAS (Qin et al., 2022) comprising 473 microbial features (*n* = 5,959), and the Dutch Microbiome Project GWAS (Lopera-Maya et al., 2022) of 205 inferred microbial metabolic pathways (*n* = 7,738). Together, these datasets capture GM variation at taxonomic and functional levels. GWAS summary statistics for circulating plasma metabolites were obtained from the study by Richards et al., which quantified 1,400 plasma metabolic traits, including 1,091 metabolites and 309 metabolite ratios, in 8,299 individuals (Chen Y. et al., 2023). The GWAS catalogue (GCST199621- GCST201020) provides an overview of the GWAS statistical data for each serum metabolite characteristic. Outcome data for DN were derived from the FinnGen consortium (Kurki et al., 2023) (release R8). Additional DN-related phenotypes included estimated glomerular filtration rate (eGFR; *n* = 11,529) and urinary albumin-to-creatinine ratio (UACR; *n* = 5,825), which were obtained from CKDGen consortium meta-analyses and accessed through the IEU OpenGWAS database (Teumer et al., 2016; Pattaro et al., 2012; Pattaro et al., 2016). All GWAS datasets included participants of predominantly European ancestry. Therefore, the MR estimates primarily reflect genetic evidence derived from European-ancestry populations. All MR analyses were based on publicly available summary-level GWAS data. Therefore, individual-level participant overlap between exposure and outcome datasets could not be directly assessed.

### Instrument variable selection

Valid instrumental variables (IVs) must satisfy the three core assumptions: (1) IVs must be reliably associated with exposure; (2) IVs must be independent of any confounders; (3) IVs must be related to outcome only via exposure. A series of procedures were applied to select single-nucleotide polymorphisms (SNPs) as IVs. Genetic instruments were selected based on their associations with each exposure in the corresponding GWAS. To ensure a sufficient number of instruments for GM traits, single-nucleotide polymorphisms (SNPs) associated at a significance threshold of *p* < 1 × 10^−5^ were selected. For all other exposures, a conventional genome-wide significance threshold (*p* < 5 × 10^−8^) was applied. To obtain independent instruments, linkage disequilibrium (LD) clumping was performed using a European reference panel, with an r^2^ threshold of < 0.001 within a 10,000 kb window. This approach minimized correlation between selected variants and reduced the potential impact of pleiotropy. Instrument strength was assessed using the F-statistic. The proportion of variance explained (R^2^) for each instrument was calculated based on the reported effect size, effect allele frequency, standard error, and sample size. SNPs with an F-statistic < 10 were excluded to reduce bias due to weak instruments (Burgess et al., 2011). Detailed information on the selected instruments and F-statistics is provided in Supplementary Tables 1–3. Before MR analysis, exposure and outcome GWAS summary statistics were harmonised to ensure that SNP effects corresponded to the same effect allele. For palindromic SNPs with A/T or C/G alleles, allele frequency information was used to infer strand orientation when available. Ambiguous palindromic SNPs for which strand orientation could not be reliably determined were removed during harmonisation. To improve transparency, harmonised SNP-level information for the reported MR associations, including effect allele, other allele, effect allele frequency, palindromic status, ambiguous status, harmonisation action, MR inclusion status, and SNP retention status, is provided in Supplementary Tables 4–7.

### Sensitivity analysis

Heterogeneity across SNP-specific estimates was assessed using Cochran’s Q test, with *p* < 0.05 indicating potential heterogeneity. Directional horizontal pleiotropy was evaluated using the MR-Egger intercept test and the MR-PRESSO global test. When MR-PRESSO detected potential outlier variants, outlier information was recorded and outlier-corrected estimates were considered when available. Leave-one-out analysis was performed to evaluate whether the overall MR estimate was driven by a single influential SNP.

Complete sensitivity results for the reported MR associations are provided in Supplementary Tables 8–11, including Cochran’s Q *p* value, MR-Egger intercept, MR-Egger intercept *p* value, MR-PRESSO global test *p* value, MR-PRESSO outlier SNPs, leave-one-out beta range, leave-one-out *p* value range, number of leave-one-out *p* values ≥ 0.05, and the most influential SNP. Associations showing heterogeneity, potential horizontal pleiotropy, or reduced leave-one-out stability were interpreted cautiously. All statistical analyses were performed using the TwoSampleMR and MR-PRESSO packages in R. These sensitivity analyses were used to evaluate the robustness of the MR estimates and to guide cautious interpretation of associations showing potential instability.

### Mendelian randomization analysis

MR analyses were performed using the inverse-variance weighted (IVW) method as the primary approach, complemented by MR-Egger regression, weighted median, simple mode, and weighted mode methods. Consistency of effect direction across multiple methods was used to support the robustness of causal estimates. Potential horizontal pleiotropy was assessed using the MR-Egger intercept and the MR-PRESSO global test. Cochran’s Q statistic was used to evaluate heterogeneity among instrumental variables. Leave-one-out analyses were conducted to examine the influence of individual SNPs and to assess the stability of the MR estimates. For associations showing consistent effect directions and no evidence of substantial pleiotropy or heterogeneity, IVW estimates were considered the primary results and were used for downstream analyses and visualization. Bidirectional MR analyses were performed to evaluate potential reverse causation among GM traits, circulating plasma metabolites, and DN phenotype. Associations with evidence of reverse causality were excluded from subsequent analyses. Given the large number of gut microbiota, microbial pathway, and plasma metabolite traits tested, false discovery rate (FDR) correction was applied to the main MR results. Associations with FDR-adjusted *p* < 0.05 were considered statistically significant. Associations with nominal *p* < 0.05 but FDR-adjusted *p* ≥ 0.05 were considered suggestive and interpreted cautiously, particularly when sensitivity analyses indicated potential heterogeneity, horizontal pleiotropy, or reduced leave-one-out stability.

### Mediation analysis

Only combinations of GM traits and plasma metabolites with consistent causal associations from the bidirectional MR analyses were included in the mediation analyses. A two-step MR framework was applied. In the first step, causal effects of GM traits on plasma metabolites were estimated. In the second step, causal effects of plasma metabolites on DN were assessed. The indirect effect of each GM trait on DN through the plasma metabolites was calculated as the product of the causal effect of the GM trait on the metabolite and the causal effect of the metabolite on DN. The direct effect was derived by subtracting the indirect effect from the total effect of the GM trait on DN. The proportion mediated was calculated as the ratio of the indirect effect to the total effect. Statistical significance was evaluated using two-sided tests. All analyses were performed using harmonized GWAS summary statistics.

### Colocalization analysis

To investigate whether GM, plasma metabolites, and DN share common genetic variants, we applied a Bayesian colocalization model. This approach estimates the posterior probability that two traits in the same genomic region may share a causal variant. The posterior probability of colocalization (PP. H4) was used to assess shared genetic signals. A PP. H4 value > 0.5 was used as a suggestive threshold to prioritize candidate loci with possible shared genetic signals. Because this threshold provides moderate rather than definitive evidence of colocalization, the results were interpreted as exploratory and considered together with MR, mediation, clinical validation, and experimental findings. Colocalization analyses were performed for the genetic associations between GM and metabolites, as well as between metabolites and DN. For trait pairs with suggestive colocalization results, we further identified candidate variants potentially associated with the DN-related pathway.

To refine the identification of these variants, we implemented a multi-step genetic locus selection strategy. SNPs associated with the mediated pathways were first selected as candidate instruments. Genomic regions around these SNPs were then extended by ±500 kb to capture candidate variants within the surrounding genetic regions. The intersection of multiple extended regions was used to define a candidate core region relevant to the pathway linking GM, metabolites, and DN. Within this core region, LD clustering was performed using an r^2^ threshold > 0.5 and a genetic distance < 0.2. SNPs in strong LD were grouped into LD blocks, which were subsequently analyzed to assess their associations with GM traits, metabolites, and DN. This approach allowed us to explore how multiple genetic signals within the same genomic region may be related to different components of the candidate GM-metabolite-DN pathway.

### Detection of blood metabolites

Healthy controls, patients with diabetes mellitus without diabetic nephropathy, and patients with biopsy-confirmed DN were recruited from a Chinese clinical cohort. The diabetes-only group was included as a disease-control group to help distinguish diabetes-related metabolic changes from DN-related changes. After an overnight fast of at least 8 h, 5 mL of venous blood was collected from the median cubital vein by trained personnel. Inclusion criteria for the DN group were age 18–75 years and biopsy-confirmed DN. Exclusion criteria were: (1) renal biopsy showing other renal pathologies in addition to DN. (2) end-stage renal disease requiring peritoneal dialysis or hemodialysis. Subjects were included if they met the diagnostic criteria for DM established by the American Diabetes Association criteria defined as fasting plasma glucose ≥ 7.0 mmoL/L, 2-h plasma glucose ≥ 11.1 mmoL/L during an oral glucose tolerance test, or random plasma glucose ≥ 11.1 mmoL/L with typical hyperglycemic symptoms; aged between 18 and 75 years old; with glycated hemoglobin levels ranging from 6.5 to 12%. Exclusion criteria were gestational diabetes or other special types of diabetes. Healthy controls were individuals who underwent routine health examinations at our hospital’s health management center during the same period and had normal results. Written informed consent was obtained from all participants. Plasma lipidomics was performed using LC-ESI-MS/MS (QTRAP® 6,500+; Sciex). The detailed parameters and quality control protocols for LC–MS/MS are provided in Supplementary methods. Baseline characteristics and candidate metabolites were compared among the three groups. Continuous variables were presented as median (interquartile range) and compared using the Kruskal-Wallis test. Categorical variables were presented as *n*/*N* (%) and compared using the chi-square test or Fisher’s exact test, as appropriate. Pairwise comparisons were performed with multiple-testing correction where applicable, and adjusted *p* values were reported for *post hoc* group comparisons.

### Detection of plasma SM

Plasma SM levels were quantified using a colorimetric assay kit (10,009,928, Cayman) according to the manufacturer’s instructions. Briefly, 10 μL plasma was added to each well, followed by the assay reaction mixture containing SM enzyme, sphingomyelinase, phosphatase, and detection buffer. After 1 h incubation at room temperature, absorbance was measured at 585–600 nm (see details in the Supplementary methods).

### Cell culture and Western blotting

Human podocytes were cultured under growth-permissive conditions at 33 °C in RPMI 1640 medium supplemented with 10% fetal bovine serum, 100 U/mL penicillin, and 100 μg/mL streptomycin. To induce differentiation, podocytes were maintained at 37 °C for 7 days before experiments. For the formal intervention experiment, differentiated podocytes were treated for 48 h under the following conditions: normal glucose (NG, 5.6 mM glucose), osmotic control (Mannitol, 5.6 mM glucose plus 24.4 mM mannitol), high glucose (HG, 30 mM glucose), vehicle control (Eth, 30 mM glucose plus 1‰ ethanol), SM200 (30 mM glucose plus 200 μg/mL sphingomyelin), and SM250 (30 mM glucose plus 250 μg/mL sphingomyelin). D-(+)-glucose was purchased from Sigma-Aldrich (cat. no. G8769), and sphingomyelin was purchased from MedChemExpress (cat. no. HY-113498). Sphingomyelin was prepared as a 300 mg/mL stock solution in ethanol and diluted into culture medium immediately before treatment. The Eth group contained 1‰ ethanol, corresponding to the ethanol concentration used for the highest sphingomyelin concentration tested in the CCK-8 assay.

### Cell counting kit-8 (CCK-8)

Human podocytes were seeded in 96-well plates at an optimized density. Cells were treated for 48 h under different conditions, including normal glucose (NG, 5.6 mM glucose), high glucose (HG, 30 mM glucose), vehicle control (Eth, 30 mM glucose plus 1‰ ethanol), and sphingomyelin treatment at 100, 150, 200, 250, and 300 μg/mL under high-glucose conditions. Cell viability was then assessed using a CCK-8 assay kit according to the manufacturer’s instructions. The original treatment medium was discarded, and cell surfaces were gently rinsed twice with PBS. Residual PBS was completely aspirated to avoid interference. Then, 110 μL of CCK-8 working solution was added to each well, and the plate was incubated at 37 °C with 5% CO₂ for 30 min. Finally, absorbance at 450 nm was measured using a microplate reader, with blank controls containing medium and CCK-8 reagent only used for background correction. Cell viability was calculated relative to the NG group.

### Western blot

After treatment, human podocytes were washed twice with cold PBS and lysed in RIPA buffer containing 1 × protease inhibitor cocktail and 1 × phosphatase inhibitor. Cell lysates were incubated on ice for 20–30 min and then centrifuged at 12,000 × g for 15 min at 4 °C. The supernatants were collected, and protein concentrations were determined using a BCA protein assay kit. Equal amounts of protein were mixed with SDS-PAGE loading buffer and heated at 99 °C for 10 min. Proteins were separated using 4–20% SDS-PAGE gels and transferred onto PVDF membranes. The membranes were blocked with 5% non-fat milk in TBST for 1–1.5 h at room temperature and then incubated with primary antibodies at 4 °C overnight. After washing with TBST, the membranes were incubated with HRP-conjugated secondary antibodies for 1 h at room temperature. Protein bands were visualized using an enhanced chemiluminescence detection system. The following primary antibodies were used: Nephrin (1:1000, cat. no. AF7951, Affinity), Podocin (1:500, cat. no. 20384-1-AP, Proteintech), FN (1:1000, cat. no. ET1702-25, Huabio), COL1A2 (1:1000, cat. no. 66761-1-Ig, Proteintech), NLRP3 (1:1000, cat. no. #15101, Cell Signaling), TNF-*α* (1:1000,cat. no. #11948, Cell Signaling), and GAPDH (1:1000, cat. no. GB11002-100, Servicebio). GAPDH was used as the internal control. Band intensities were quantified using ImageJ software and normalized to GAPDH. Western blot experiments were performed with three independent biological replicates.

### Ethical approval and informed consent

All GWAS summary statistics used in this study were obtained from publicly available repositories. The original investigations that generated these data adhered to the principles of the World Medical Association’s Declaration of Helsinki and obtained approval from institutional review boards (IRB) or ethics committees, as well as informed consent from the participants. As this work is a secondary analysis of fully de-identified, aggregate data with no new recruitment or interventions, no additional ethical approval or consent was required for the present study. All the related procedures for human samples were performed with the Declaration of Helsinki and approved by Ethics Committee of the Zhengzhou University People’s Hospital (Approval No. 2020207).

## Results

### Identification of GM taxa and pathways with MR-supported potential causal associations with DN-related phenotypes

**Figure 2:**
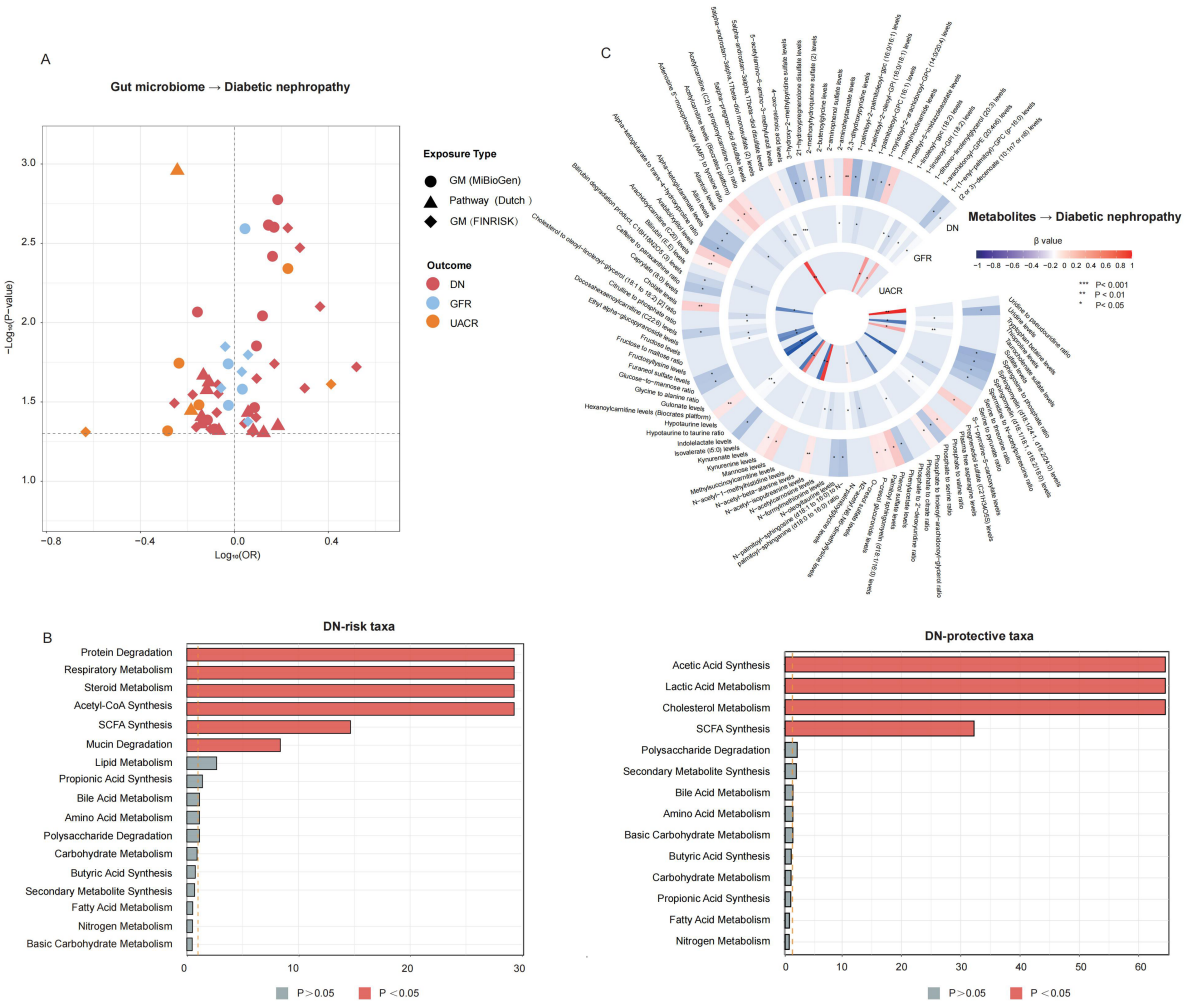
Causal associations between GM, plasma metabolites and DN phenotypes (GM/metabolites to DN). **(A)** The volcano plot displays causal relationships between DN and GM, with dot shapes denoting exposure categories and colors representing different DN phenotypes as outcomes. Dashed lines indicate *p*-value (*p* < 0.05) and odds ratio (OR = 1) thresholds. **(B)** The enrichment analysis of DN-related GM. **(C)** The circular heatmap illustrates causal relationships identified in the MR analysis showing associations between metabolites and DN phenotypes. The inner circle displays associations between metabolites and both eGFR and UACR, while the outer circle highlights associations with DN. Cells are colored by beta coefficients: red for positive and blue for reverse associations. Significance levels are indicated as *p < 0.05; **p < 0.01; ***p < 0.001.

Bidirectional MR analyses were conducted to evaluate potential causal relationships between three GM datasets and DN-related phenotypes. Forward MR identified multiple GM traits associated with these outcomes. After excluding associations showing evidence of reverse causality, 25 species, 22 higher-level taxa spanning genus to phylum levels, and 12 microbial metabolic pathways were significantly associated with at least one DN-related phenotype (Figure 2A; Supplementary Figure 1). At the species level, *Omnitrophota*, *Aureimonas*, and *Poseidoniaceae* were positively associated with DN, whereas *Emergencia* and *Bin127* were inversely associated. Among higher-level taxa, the order *Bacteroidales*, family *Verrucomicrobiae*, class *Bacteroidales*, and genus *Parasutterella* were positively linked to DN-related phenotypes, while the family *Peptostreptococcaceae* and the species *Holdemania* were inversely correlated. Regarding microbial pathway profiles, the preQ0 biosynthesis pathway was inversely associated with UACR, whereas the pentose phosphate pathway, non-oxidative branch, was positively correlated with DN. To explore the functional relevance of GM in DN, we focused on taxa showing causal associations and performed functional enrichment analysis. Pathways involved in lipid metabolism and short-chain fatty acid (SCFA) synthesis were significantly enriched (Figure 2B). The complete MR results with FDR-adjusted *p* values are provided in Supplementary Tables 12–15.

### Identification of plasma metabolites with MR-supported potential causal associations with DN-related phenotypes

A total of 152 plasma metabolites showed nominal associations with DN-related phenotypes. Several lipid metabolism-related plasma metabolites showed nominal associations with DN-related phenotypes. These included acetylcarnitine levels, kynurenine levels, palmitoyl sphingomyelin (d18:1/16:0) levels, sphingosine to phosphate ratio, and sphingomyelin (d18:1/24:1 and d18:2/24:0) levels. Amino acid-related metabolites, including tryptophan betaine levels and the citrulline to phosphate ratio, were significantly associated with eGFR. Additionally, glucose metabolism-related metabolites, such as fructosyllysine levels, were associated with UACR (Figure 2C). To identify the physiological processes associated with the blood metabolites, we performed an enrichment analysis using the MetaboAnalyst database. The enrichment analysis revealed that these 152 metabolites are involved in processes such as the degradation of fructose and mannose metabolism, galactose metabolism, and others (Supplementary Figure 2).

### Sensitivity analyses of MR results

Sensitivity analyses were performed for the reported MR associations and are summarized in Supplementary Tables 8–11. Most reported associations showed no strong evidence of directional horizontal pleiotropy according to MR-Egger intercept and MR-PRESSO global tests. Leave-one-out analysis suggested that the main findings were generally not driven by a single SNP. Associations showing heterogeneity, potential pleiotropy, or reduced leave-one-out stability were explicitly flagged and interpreted cautiously.

### Bidirectional relationships between GM and plasma metabolites

**Figure 3:**
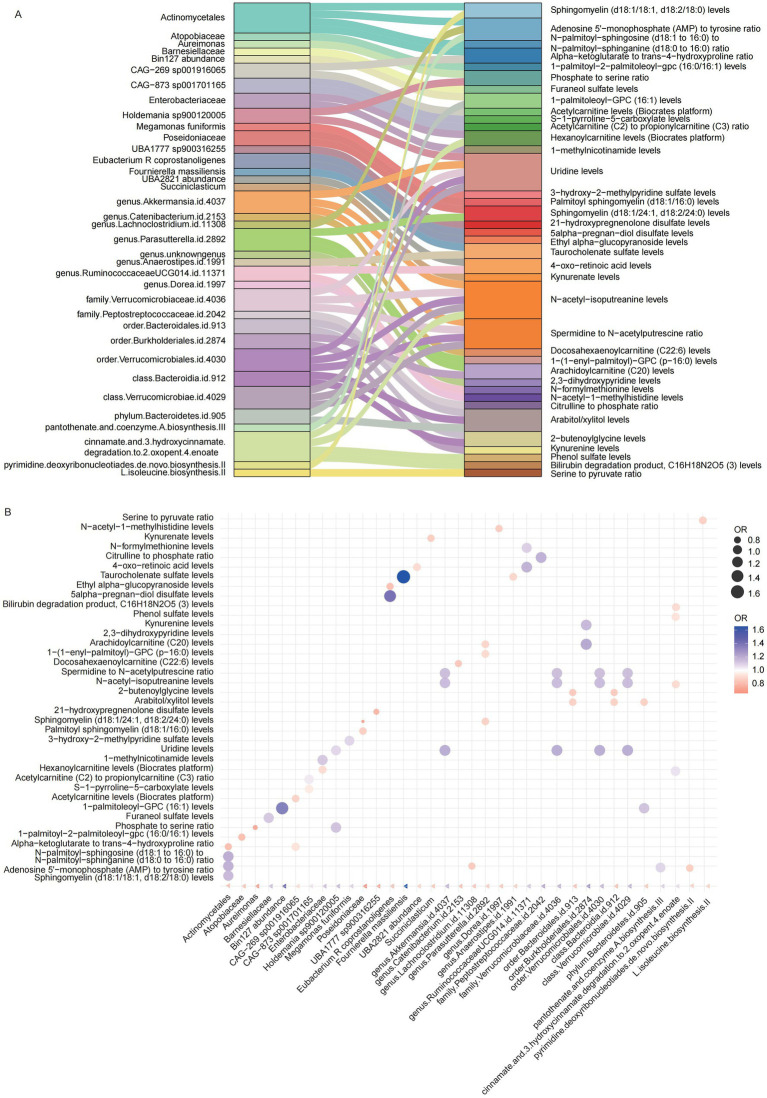
Causal associations between gut microbiota and plasma metabolites. **(A)** Sankey diagram: Left nodes denote gut microbiota, right nodes denote plasma metabolites, and connecting lines indicate their potential associations. **(B)** Causal relationship forest plot: The ordinate represents plasma metabolites (consistent with those in the Sankey diagram), and the abscissa represents the OR with 1.0 as the reference. An *OR* > 1.0 indicates a potential positive effect of the corresponding GM on plasma metabolite levels, while an *OR* < 1.0 indicates a potential inverse effect. Error bars reflect confidence intervals, illustrating the direction and relative strength of the causal associations.

Following the bidirectional MR analyses of GM traits and plasma metabolites with DN-related phenotypes, we identified several microbiota traits and metabolites showing MR-supported potential causal associations with DN, eGFR, and UACR. We then performed bidirectional MR to assess potential causal relationships between GM traits and plasma metabolites that had shown MR-supported associations with DN-related phenotypes in the preceding analyses. The analysis suggested potential causal effects of the order Verrucomicrobiales and the genus Parasutterella on multiple plasma metabolites. Conversely, metabolites such as the spermidine to N-acetylputrescine ratio and N-acetyl-isoputreanine levels also showed MR-supported potential causal associations with various GM features. These multi-to-multi relationships highlight complex, layered interactions between GM and metabolites, suggesting potential reciprocal regulatory mechanisms linking GM, metabolites, and DN (Figures 3A,B). The complete bidirectional MR results for DN-, eGFR-, and UACR-related microbiota-metabolite pairs are provided in Supplementary Tables 16–18.

### Mediation analysis prioritizes candidate metabolite-mediated pathways

Bidirectional MR analyses identified 51 GM-metabolite pairs with MR-supported potential causal relationships. Among these, 38 were associated with DN, 10 with eGFR, and 3 with UACR. Subsequent mediation analyses prioritized three candidate pathways in which plasma metabolites may mediate the potential causal effects of GM on DN.

**Figure 4:**
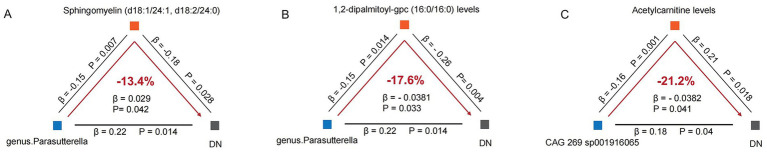
Identification of candidate mediating metabolites between gut microbiota and DN phenotypes using mediation analysis. **(A)** Genus *Parasutterella*-sphingomyelin (d18:1/24:1, d18:2/24:0)-DN. **(B)** Genus *Parasutterella*-1,2-dipalmitoyl-GPC (16:0/16:0)-DN. **(C)** CAG-269 sp001916065-acetylcarnitine-DN.

Genetically predicted genus *Parasutterella* abundance showed a potential causal effect on sphingomyelin (d18:1/24:1, d18:2/24:0) levels, and mediation analysis suggested that this metabolite may partially mediate the effect of genus *Parasutterella* on DN (Figure 4A). The MR-estimated total causal effect of genus *Parasutterella* abundance on DN risk was positive (*β* = 0.22, *p* = 0.01). Mediation analysis indicated a significant indirect effect of 0.029 (95% *CI*: 0.000949–0.0571, *p* = 0.042), accounting for 13.4% of the total effect (95% *CI*: 0.439–26.4%). Directional analyses showed that increased genus *Parasutterella* abundance was associated with reduced sphingomyelin (d18:1/24:1, d18:2/24:0) levels (*OR* = 0.85, *β* = −0.18, *p* = 0.007), while genetically predicted higher levels of this specific sphingomyelin signal were associated with a reduced risk of DN (*OR* = 0.83, *β* = −0.18, *p* = 0.028). These findings suggest a suppressive mediation pattern in which genetically predicted sphingomyelin (d18:1/24:1, d18:2/24:0) was inversely associated with DN risk. Therefore, this result should be interpreted as a species-specific MR signal rather than evidence that total sphingomyelin or all sphingomyelin species are protective. Overall, the estimated mediated proportion was 13.4%, suggesting that sphingomyelin (d18:1/24:1, d18:2/24:0) may partly mediate the MR-supported association between genus Parasutterella and DN, whereas the remaining association may reflect other microbial-related or unmeasured pathways.

In addition, 1,2-dipalmitoyl-GPC (16:0/16:0) was identified as another mediator linking genus *Parasutterella* to DN (Figure 4B). The indirect effect through this metabolite was −0.0381 (95% *CI*: −0.0731 to −0.00298, *p* = 0.033), corresponding to a mediated proportion of −17.6% (95% *CI*: −33.8 to −1.38%). This negative mediated proportion indicates a suppressive mediation pattern, in which the metabolite exerted a protective indirect effect that partially offset the direct risk effect of *Parasutterella* (*β* = 0.2581). Despite this attenuation, the total effect remained a net risk effect, driven primarily by the direct pathway.

A similar suppressive mediation pattern was observed for CAG-269 sp001916065 (Figure 4C). The total effect of CAG-269 sp001916065 abundance on DN risk was positive (*OR* = 1.20, *β* = 0.18, *p* < 0.05). Acetylcarnitine levels mediated this association, with an indirect effect of −0.0382 (95% *CI*: −0.075 to −0.00152, *p* = 0.041), accounting for −21.2% of the total effect (95% *CI*: −41.5 to −0.843%). Directional analyses indicated that CAG-269 sp001916065 abundance was associated with reduced acetylcarnitine levels (*OR* = 0.85, *β* = −0.16), while acetylcarnitine exerted a protective effect on DN risk. As with genus *Parasutterella*, the direct effect of the microbiota remained a risk factor, resulting in an overall suppressive mediation model. The detailed mediation effects are provided in Supplementary Table S19.

### Colocalization analysis of GM, metabolites, and DN

**Figure 5:**
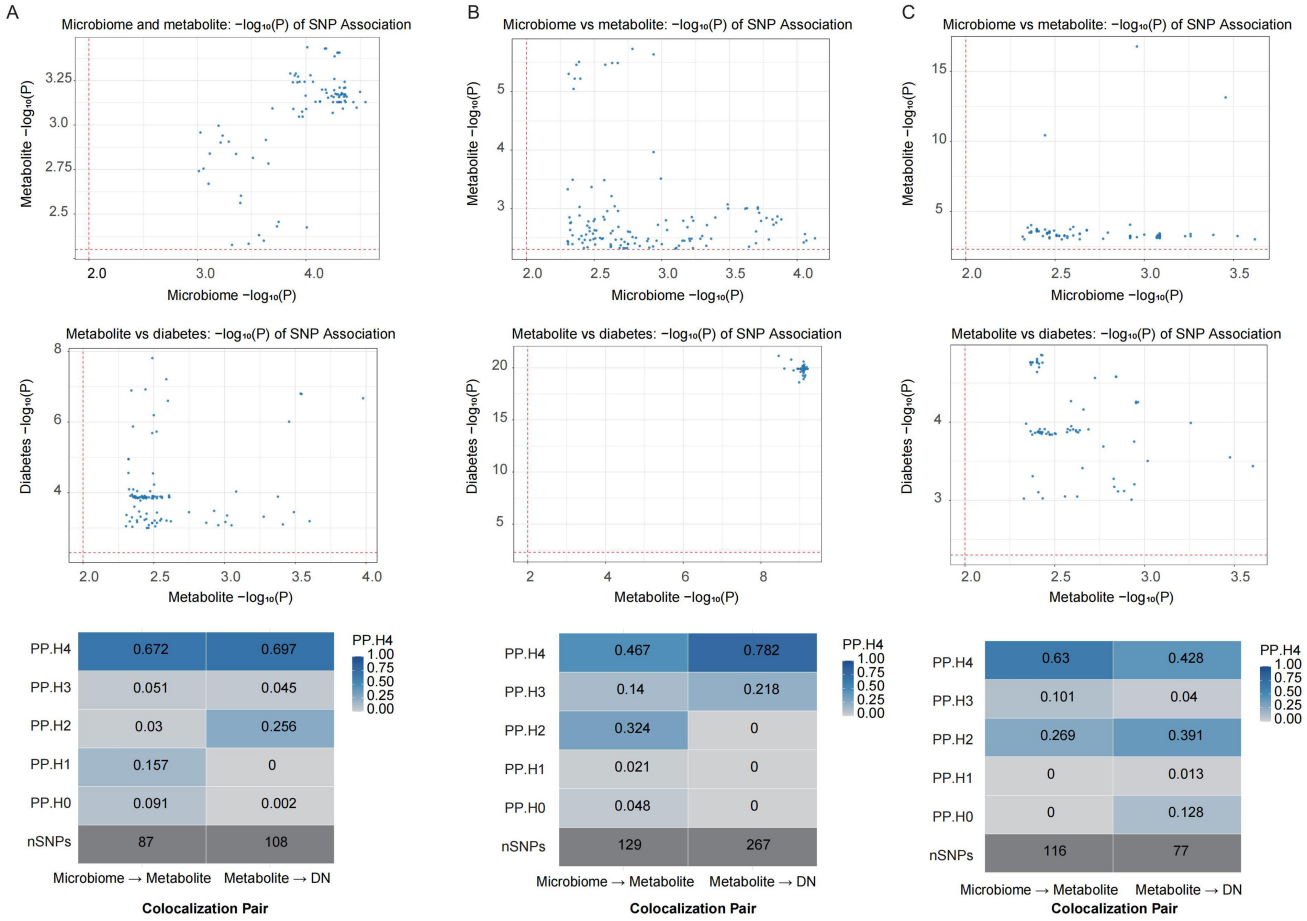
SNP association and exploratory colocalization analysis of gut microbiota, plasma metabolites, and diabetic nephropathy. Scatter plots (upper panels) show the −log₁₀(P) values of SNP associations between gut microbiota and plasma metabolites. Scatter plots (middle panels) show the −log₁₀(P) values of SNP associations between plasma metabolites and diabetic nephropathy. Corresponding tables (lower panels) present key parameters for the SNP association and exploratory colocalization analyses: nSNP (number of SNPs), PP. H0-PP. H4 (posterior probabilities of five colocalization hypotheses). The x − axis and y-axis represent −log₁₀(P) values of the respective SNP associations, with higher values indicating stronger association significance. **(A)** SNP association analysis of genus *Parasutterella*, sphingomyelin (d18:1/24:1, d18:2/24:0) levels, and DN. **(B)** SNP association analysis of genus *Parasutterella*, 1,2-dipalmitoyl-GPC (16:0/16:0), and DN. **(C)** SNP association analysis of CAG-269 sp001916065, acetylcarnitine, and DN. Micr, gut microbiota; Meta, plasma metabolites; DN, diabetic nephropathy.

Bayesian colocalization analysis provided suggestive evidence of possible shared genetic signals for the pathway involving genus *Parasutterella*, sphingomyelin (d18:1/24:1, d18:2/24:0), and DN, with *PP. H4* values of 0.672 for the microbiome-metabolite association and 0.697 for the metabolite-DN association (Figure 5A). In contrast, the other two candidate pathways showed inconsistent colocalization support: only one of the two tested associations had a PP. H4 value above 0.5 in each pathway (Figures 5B,C).

**Figure 6:**
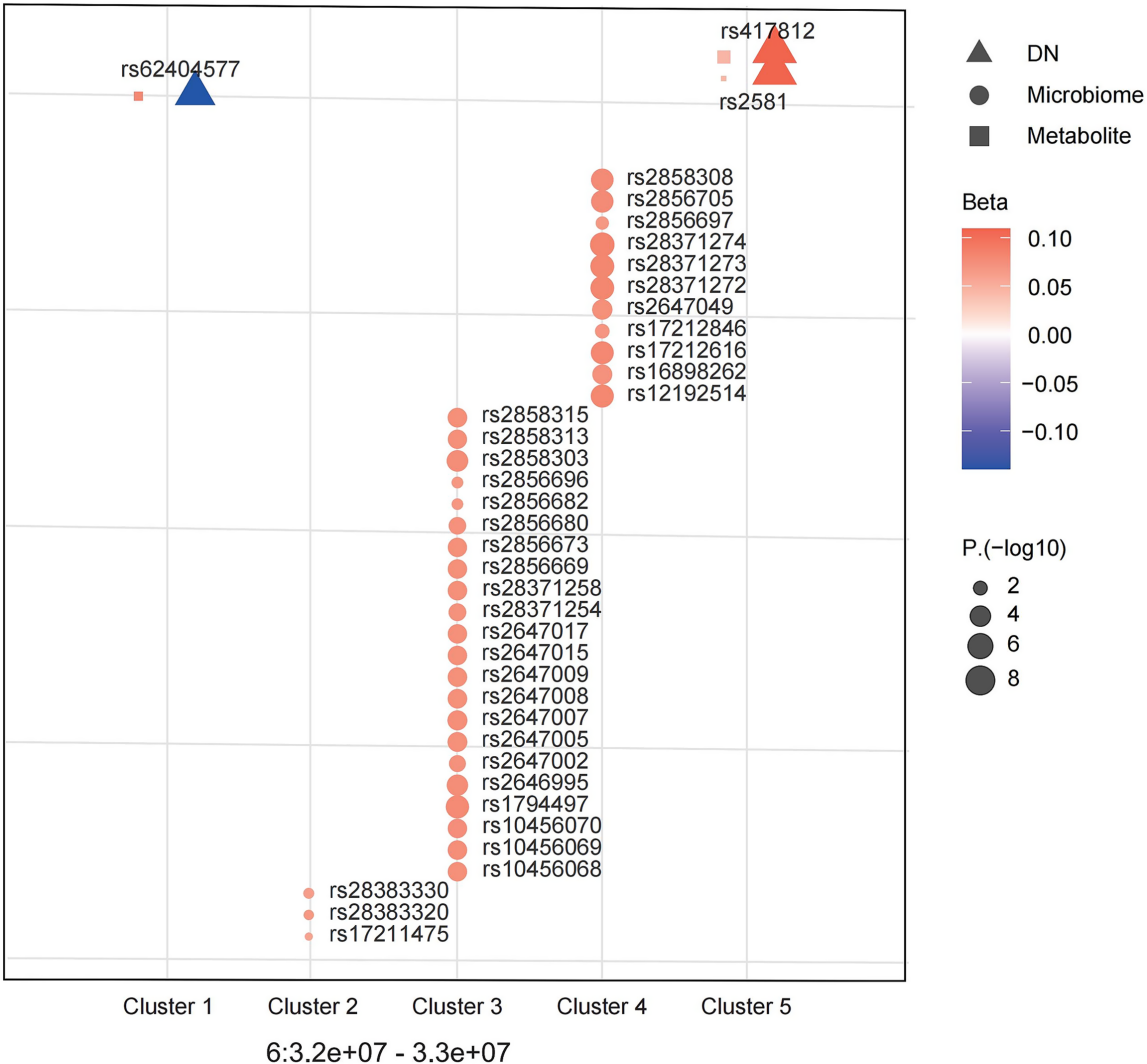
Association characteristics of SNPs related to gut microbiota, plasma metabolites, and DN within a specific chromosomal region (chromosome 6: 3.2e+07–3.3e+07).

To further explore the location of candidate genetic signals, we expanded the genomic regions surrounding the instrumental SNPs in the sphingomyelin pathway by 500 kilobases on each side, creating candidate intervals of 1 megabase. The overlapping segments defined a candidate core locus on chromosome 6, spanning 32 to 33 megabases. LD clustering within this region revealed a clear separation of genetic signals along the pathway. Variants associated with genus *Parasutterella* were primarily concentrated in clusters 2, 3, and 4, which included 36 SNPs with mean *p* values between 0.0011 and 0.0041. By contrast, associations between sphingomyelin (d18:1/24:1, d18:2/24:0) levels and DN were mainly found in clusters 1 and 5, consisting of three SNPs with metabolite *p* values ranging from 0.0039 to 0.0044 and DN *p* values from 0.0438 to 0.0789 (Figure 6).

When considering all SNPs within the candidate core locus, association signals were observed for genus *Parasutterella*, sphingomyelin (d18:1/24:1, d18:2/24:0) levels, and DN, with mean *p* values of 0.00159, 0.00413, and 4.56 times 10 to the minus five, respectively. The directions of the effects were generally concordant between microbiota-associated and metabolite-associated variants, providing exploratory support for prioritizing a potential Parasutterella-sphingomyelin-DN pathway. Overall, these findings suggest that the chromosome 6 locus may contain genetic signals relevant to GM, sphingolipid metabolites, and DN. These findings do not support a single shared variant as the sole explanation; rather, multiple LD blocks may contribute to the observed association pattern across different components of the candidate pathway. Functional annotation of variants in this region highlighted three nearby genes: *MTCO3P1*, *HLA-DOA*, and *BTNL2*.

### Detection of identified blood metabolites and clinical correlation analysis

**Figure 7:**
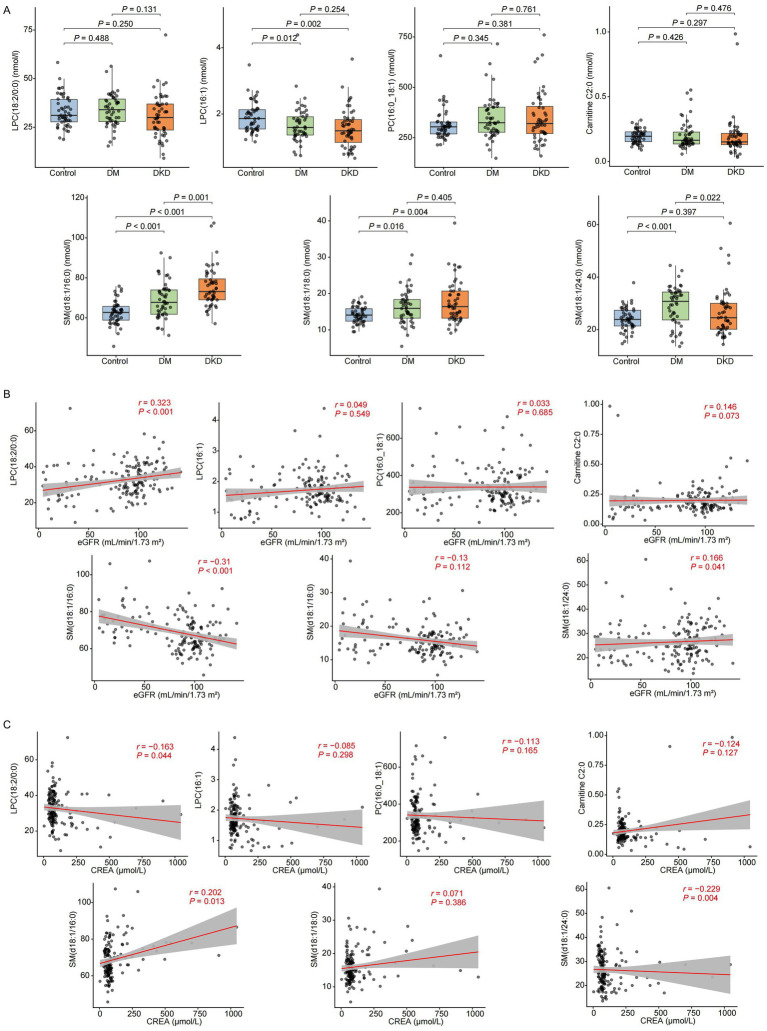
Metabolic profiles and their associations with kidney function in the clinical validation cohort. **(A)** Boxplots comparing the levels of seven candidate metabolites among healthy controls, diabetes-only patients, and DN patients. Error bars represent the standard deviation. **(B)** Scatter plot showing the correlation between the levels of the seven metabolites and eGFR in DN patients. **(C)** Scatter plots showing correlations between the seven candidate metabolites and eGFR/serum creatinine in the overall clinical cohort. Group comparisons were performed using the Kruskal-Wallis test followed by multiple-testing-corrected pairwise comparisons where applicable. Correlations were assessed using Spearman correlation analysis. *p < 0.05; **p < 0.01; ***p < 0.001; ns, not significant.

We performed plasma metabolomics in healthy controls (*n* = 50), patients with diabetes mellitus without DN (*n* = 50), and patients with biopsy-confirmed DN (*n* = 52). Baseline clinical and laboratory characteristics of the three groups are summarized in Table 1. Age, sex, and BMI were comparable among healthy controls, diabetes-only patients, and patients with biopsy-confirmed DN. Compared with diabetes-only patients, patients with DN showed more severe renal dysfunction, including higher serum urea and creatinine levels and lower eGFR. Traditional lipid profiles, including total cholesterol, triglycerides, HDL-C, LDL-C, and non-HDL-C, as well as the proportions of lipid-lowering medication and statin use, were not significantly different between the diabetes-only and DN groups. We intersected these data with the 152 metabolites linked to DN phenotype in the prior MR analysis. Seven metabolites were detected: Carnitine C2:0, LPC (16:1), LPC (18:2/0:0), PC (16:0_18:1), SM (d18:1/16:0), SM (d18:1/24:0), and SM (d18:1/18:0). The seven MR-prioritized candidate metabolites were compared among the three groups. Among them, SM (d18:1/16:0) showed a progressive increase from healthy controls to diabetes-only patients and DN patients, and was significantly higher in DN patients than in diabetes-only patients (Figure 7A). To assess whether these differential metabolites reflect renal function decline in DN, we measured eGFR and serum creatinine (CREA) and tested correlations for the seven metabolites. As shown, SM (d18:1/16:0) (*r* = −0.31, *p* < 0.001) was negatively correlated with eGFR, while LPC (18:2/0:0) was positively correlated with eGFR (*r* = 0.323, *p* < 0.001) (Figure 7B). In correlation analyses with CREA, LPC (18:2/0:0) (*r* = −0.163, *p* = 0.044) and SM (d18:1/24:0) (*r* = −0.229, *p* = 0.004) showed negative correlations, whereas SM (d18:1/16:0) showed a positive correlation with CREA (*r* = 0.202, *p* = 0.013) (Figure 7C). These findings suggest that several metabolites, particularly sphingomyelin-related species, may serve as candidate biomarkers for diabetic nephropathy and support further studies on sphingomyelin remodeling in DN. Together, these clinical metabolomic results provide preliminary patient-level support for sphingomyelin dysregulation in DN, while independent validation and direct assessment of genus Parasutterella abundance require further clinical cohorts with matched microbiome and metabolomic data.

**Table 1:** Baseline clinical and laboratory characteristics of three groups.

Variable	NC (*n* = 50)	DM (*n* = 50)	DKD (*n* = 52)	*P* value
Age, years	49.50 (39.00, 57.00)	52.00 (44.00, 60.00)	53.00 (45.00, 62.50)	0.170
Male sex, *n*/*N* (%)	30/50 (60.0%)	30/49 (61.2%)	28/51 (54.9%)	0.792
BMI, kg/m^2^	24.25 (23.08, 25.97)	24.65 (23.15, 26.53)	25.71 (22.77, 27.95)	0.181
Diabetes duration, years	0	5.00 (2.00, 10.00)	9.00 (4.00, 12.00)	0.087
Glucose-lowering treatment use, *n*/*N* (%)	0/50 (0.0%)	49/50 (98.0%)ᵃ	47/51 (92.2%)ᵇ	<0.001
Hypertension, *n*/*N* (%)	0/50 (0.0%)	16/48 (33.3%)ᵃ	51/52 (98.1%)ᵇᶜ	<0.001
Antihypertensive treatment use, *n*/*N* (%)	0/50 (0.0%)	16/48 (33.3%)ᵃ	49/51 (96.1%)ᵇᶜ	<0.001
Lipid-lowering medication use, *n*/*N* (%)	0/50 (0.0%)	33/50 (66.0%)ᵃ	27/52 (51.9%)ᵇ	<0.001
Statin use, *n*/*N* (%)	0/50 (0.0%)	32/50 (64.0%)ᵃ	26/52 (50.0%)ᵇ	<0.001
FBG, mmol/L	5.04 (4.79, 5.26)	7.88 (6.20, 9.49)ᵃ	5.72 (4.93, 7.03)ᵇᶜ	<0.001
HbA1c, %	5.25 (5.03, 5.50)	7.95 (6.73, 9.35)ᵃ	6.80 (5.93, 8.87)ᵇᶜ	<0.001
Urea, mmol/L	5.09 (4.42, 5.49)	5.79 (4.54, 7.08)ᵃ	10.18 (8.44, 16.47)ᵇᶜ	<0.001
Creatinine, μmol/L	71.65 (65.98, 83.33)	55.00 (45.17, 64.97)ᵃ	160.85 (82.50, 248.88)ᵇᶜ	<0.001
eGFR, mL/min/1.73 m^2^	96.88 (87.39, 103.12)	105.01 (97.85, 118.06)ᵃ	37.39 (20.33, 73.76)ᵇᶜ	<0.001
Total cholesterol, mmol/L	4.42 (3.98, 4.89)	4.74 (4.11, 5.45)	4.96 (4.07, 6.37)ᵇ	0.013
Triglycerides, mmol/L	1.28 (1.08, 1.59)	1.78 (1.10, 2.53)ᵃ	1.82 (1.33, 2.67)ᵇ	<0.001
HDL-C, mmol/L	1.32 (1.15, 1.56)	1.05 (0.91, 1.21)ᵃ	1.07 (0.91, 1.26)ᵇ	<0.001
LDL-C, mmol/L	2.42 (2.10, 3.00)	2.70 (2.15, 3.38)	2.68 (2.13, 3.48)	0.157
Non-HDL-C, mmol/L	3.07 (2.61, 3.54)	3.69 (2.99, 4.34)ᵃ	3.77 (3.06, 4.98)ᵇ	<0.001

Overall *p* values represent comparisons among the three groups. Superscript letters indicate significant pairwise differences after BH adjustment: ᵃ*p* < 0.05 for DM vs NC, ᵇ*p* < 0.05 for DKD vs NC, and ᶜ*p* < 0.05 for DKD vs DM.

### Experiment validation

**Figure 8:**
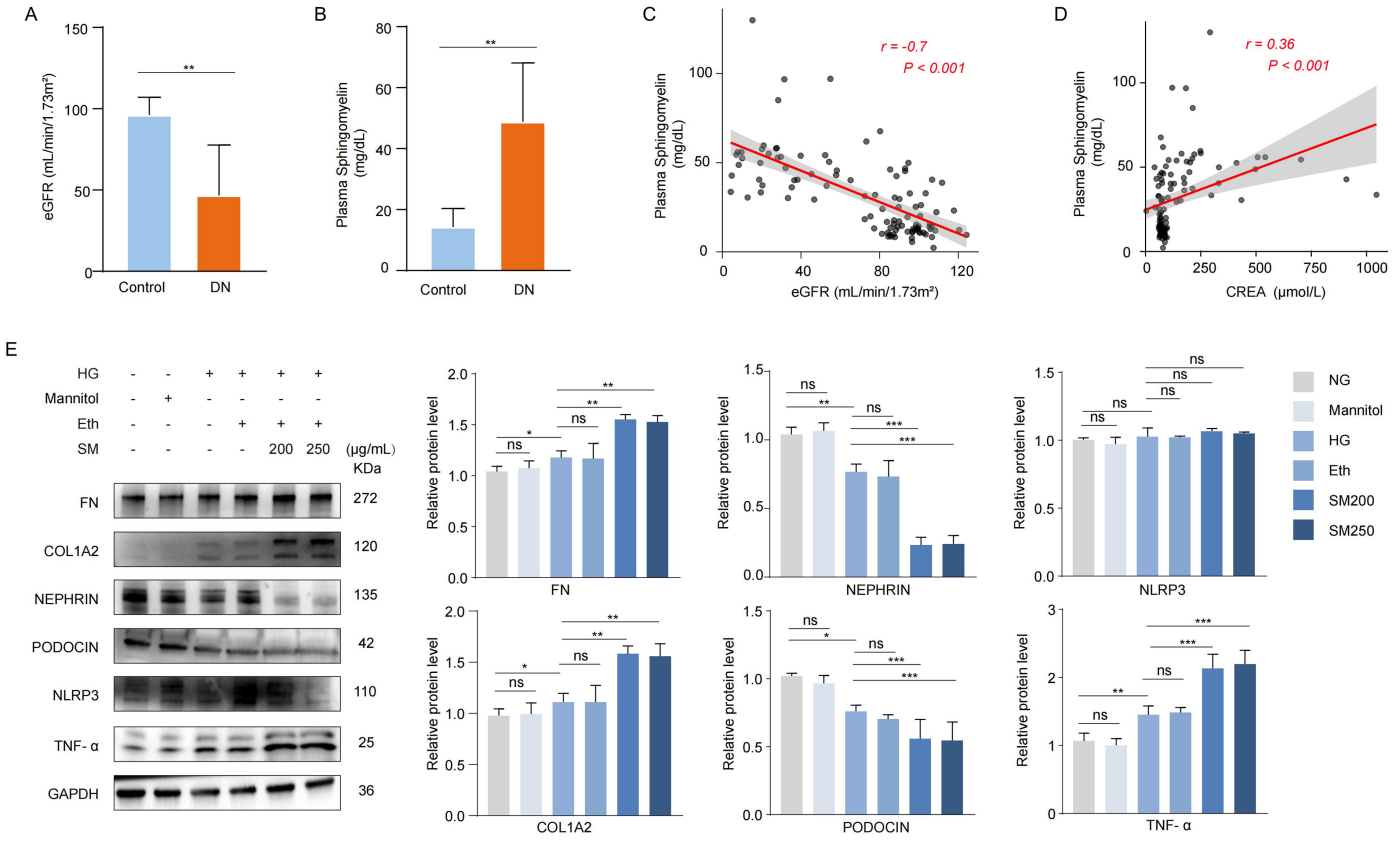
Plasma total sphingomyelin levels and sphingomyelin intervention in podocytes under high-glucose conditions. **(A)** eGFR levels in control and DN groups. **(B)** Plasma total sphingomyelin levels in control and DN groups. **(C,D)** Correlations between plasma total sphingomyelin levels and eGFR or serum creatinine. **(E)** Western blot analysis and quantification of podocyte injury-related markers Nephrin and Podocin, fibrotic/ECM-related markers FN and COL1A2, and inflammatory marker NLRP3 and TNF-α in podocytes treated with NG, Mannitol, HG, Eth, SM200, and SM250. NG, normal glucose; Mannitol, osmotic control with 5.6 mM glucose plus 24.4 mM mannitol; HG, high glucose; Eth, ethanol vehicle control; SM200, high glucose plus 200 μg/mL sphingomyelin; SM250, high glucose plus 250 μg/mL sphingomyelin. Data are presented as mean ± SD from three independent biological replicates. *p < 0.05; **p < 0.01; ***p < 0.001; ns, not significant.

Given that mediation analysis highlighted sphingomyelin as a candidate metabolic component, we further measured total plasma sphingomyelin in the clinical cohort. As expected, eGFR was significantly lower in the DN group than in the control group (Figure 8A). Total plasma sphingomyelin was markedly higher in the DN group than in the control group (control: 14.28 ± 6.09 mg/dL; DN: 48.86 ± 19.25 mg/dL; *p* < 0.001) (Figure 8B). Correlation analysis further showed that total plasma sphingomyelin was negatively correlated with eGFR (*r* = −0.70, *p* < 0.001) and positively correlated with serum creatinine (CREA) (*r* = 0.36, *p* < 0.001) (Figures 8C,D). These results suggest that plasma sphingomyelin dysregulation is associated with renal function impairment in DN. This result reflects total plasma sphingomyelin changes in established DN and should be distinguished from the species-specific sphingomyelin signal prioritized in the MR analysis. We further performed sphingomyelin intervention experiments in human podocytes.

CCK-8 assays were performed to screen appropriate sphingomyelin concentrations under high-glucose conditions. Cell viability remained above 70% at 250 μg/mL but decreased to approximately 70% at 300 μg/mL; therefore, 200 and 250 μg/mL were selected for subsequent intervention experiments (Supplementary Figure 3). For the formal intervention experiment, podocytes were assigned to six groups: NG, Mannitol, HG, Eth, SM200, and SM250. The mannitol group was used as an osmotic control, and the Eth group was used as the vehicle control. This design allowed us to distinguish high-glucose-related effects from osmotic and solvent-related effects. Western blot analysis showed that high glucose reduced the expression of podocyte injury-related proteins Nephrin and Podocin and increased the expression of fibrotic/extracellular matrix-related proteins FN and COL1A2. The mannitol group did not reproduce the same injury-related changes, and the ethanol vehicle did not markedly alter protein expression compared with HG. Compared with the vehicle control, sphingomyelin treatment under high-glucose conditions further reduced Nephrin and Podocin expression and increased FN, COL1A2, and TNF-*α* expression, especially in the SM250 group, whereas NLRP3 did not show a consistent or significant change among groups (Figure 8E). These findings suggest that sphingomyelin exposure may aggravate high-glucose-induced podocyte injury, extracellular matrix remodeling, and inflammatory-related responses.

## Discussion

To the best of our knowledge, this is one of the first studies to integrate Mendelian randomization, mediation analysis, colocalization analysis, clinical plasma metabolomics, and podocyte experiments to investigate the potential links among gut microbiota, circulating metabolites, and diabetic nephropathy. Our genetic analyses prioritized genus Parasutterella and sphingomyelin-related metabolism as a candidate pathway associated with DN. In the clinical validation cohort, we further included a diabetes-only group as a disease-control group and found that SM (d18:1/16:0) showed a progressive increase from healthy controls to diabetes-only patients and DN patients. In addition, sphingomyelin-related changes were associated with renal function indices in the overall clinical cohort, and podocyte experiments showed that sphingomyelin exposure under high-glucose conditions aggravated podocyte injury-related, fibrotic/extracellular matrix-related, and inflammatory-related changes. Together, these findings suggest that sphingomyelin metabolism may be involved in DN-related metabolic dysregulation and provide a rationale for further investigation of the genetically prioritized Parasutterella-sphingomyelin-DN pathway.

The GM is primarily composed of the phyla Firmicutes, Bacteroidetes, Actinobacteria, Proteobacteria, and Verrucomicrobia, with Firmicutes and Bacteroidetes representing approximately 60 and 15% of total abundance, respectively. Its composition varies with age, ethnicity, geography, diet, and disease status. Previous studies have shown that alterations in GM and their metabolites are involved in the development and progression of chronic kidney disease, including DN, and these changes can occur at multiple stages of the disease. Animal and human studies have demonstrated associations between microbial taxa and albuminuria levels. For example, *Prevotellaceae NK3B31* and *CAG-352* were enriched in macroalbuminuria compared to microalbuminuria groups, while *Firmicutes*, *Turicibacter*, *Syntrophococcus*, and *Akkermansia* showed altered abundance in diabetic mice compared with controls. Dietary interventions, such as fiber supplementation, also modulate gut microbial composition and are associated with changes in albuminuria, highlighting the functional relevance of microbial shifts in DN.

Our genetic analyses prioritized genus Parasutterella as a candidate microbial feature potentially related to DN, which is consistent with prior reports linking Parasutterella to metabolic disorders. Genus *Parasutterella* belongs to the family Sutterellaceae, order Burkholderiales, class Betaproteobacteria, and phylum Proteobacteria. It is an obligate anaerobic, Gram-negative, succinate-producing bacterium, previously recognized as a core genus of the healthy GM. Parasutterella contains two type strains, *Parasutterella excrementihominis* YIT11859 and *Parasutterella secunda* YIT12071 (Ju et al., 2019). Its relative abundance has been linked to multiple diseases, including irritable bowel syndrome, obesity, type 2 diabetes, and fatty liver disease. A large multicohort study found that the genus *Parasutterella*, especially *P. excrementihominis*, was positively associated with obesity and type 2 diabetes, and its abundance decreased after weight-loss interventions. Its abundance correlated positively with carbohydrate intake, particularly monosaccharides, and negatively with dietary *ω*-3 *α*-linolenic acid. Genus *Parasutterella* is a major consumer of L-cysteine and is linked to activation of host fatty acid biosynthesis. These results indicate that genus *Parasutterella* may promote obesity by linking dietary carbohydrate intake to host fatty acid biosynthesis, and may affect the development of type 2 diabetes by altering L-cysteine metabolism and glucose regulation (Henneke et al., 2022). Animal experiments showed that a low-protein diet supplemented with α-ketoacids significantly increased the abundance of genus *Parasutterella* compared with a normal-protein diet in 5/6 nephrectomized mice, suggesting that dietary interventions may modulate its levels (Zhu et al., 2022).

*Parasutterella* may also influence bile acid homeostasis and cholesterol metabolism. Colonization with *Parasutterella* altered cecal metabolites, affecting tryptophan, tyrosine, and purine metabolism. Specifically, levels of tryptophan metabolites, including 3-methyldioxyindole, indole-2-carboxylic acid, and indole-3-carboxylic acid, were increased, while kynurenic acid and nicotinic acid decreased (Feng et al., 2019). Tyrosine metabolism was similarly affected, with reduced levels of p-cresol and p-cresol sulfate, but elevated ethylphenol and a predicted derivative, N-hydroxy-L-tyrosine or DOPA (Ju et al., 2019). *Parasutterella* may also participate in glycine-conjugated metabolism in CKD rats (Feng et al., 2019). In these animals, metabolites such as myo-inositol, phenylpropionylglycine, serine, glutamine, and N1-acetylspermidine were significantly elevated and positively correlated with *Parasutterella* abundance.

Plasma metabolites, particularly sphingolipid-related metabolites, were prioritized in our MR and mediation analyses. However, sphingomyelin should not be interpreted as a single uniform molecule. In the MR analysis, the sphingomyelin-related signal was specifically sphingomyelin (d18:1/24:1, d18:2/24:0), whereas the clinical validation measured total plasma sphingomyelin and individual metabolites such as SM (d18:1/16:0), SM (d18:1/18:0), and SM (d18:1/24:0). Similarly, the podocyte experiment used a commercially obtained sphingomyelin reagent to evaluate the functional effect of sphingomyelin exposure under high-glucose conditions, rather than to directly model the MR-prioritized sphingomyelin (d18:1/24:1, d18:2/24:0) species. Therefore, the inverse association observed for genetically predicted sphingomyelin (d18:1/24:1, d18:2/24:0) should not be generalized to all sphingomyelin species or to total sphingomyelin.

Previous studies also suggest that the biological effects of sphingolipids may depend on acyl-chain length and saturation. Mäkinen et al. reported in the FinnDiane Study that serum sphingomyelin was associated with kidney disease in type 1 diabetes (Mäkinen et al., 2012). A subsequent prospective FinnDiane analysis by Pongrac Barlovic et al. (2020) further showed that higher serum sphingomyelin was associated with faster eGFR decline and adverse renal outcomes, including progression to end-stage renal disease. These findings are consistent with our observation that total plasma sphingomyelin was elevated in DN and was associated with renal dysfunction.

In contrast, other lipidomic studies have suggested that certain longer-chain or very-long-chain sphingolipid-related molecules may show different associations with kidney outcomes. Tofte et al. reported distinct associations between sphingomyelin species and renal outcomes in type 1 diabetes. In their longitudinal analyses, several longer-chain sphingomyelin species, including SM (d18:1/24:0), SM (d40:1), and SM (d41:1), were associated with a lower risk of the combined renal endpoint, and SM (d18:1/24:0) was specifically associated with a lower risk of albuminuria progression (Tofte et al., 2019). These findings suggest that not all sphingomyelin species have the same relationship with renal disease progression. Klein et al. also observed in the DCCT/EDIC cohort that decreased plasma levels of selected very-long-chain ceramide species were associated with the later development of macroalbuminuria, suggesting that relatively higher levels of these species may be linked to a lower risk of diabetic kidney disease progression (Klein et al., 2014). Although ceramides and sphingomyelins are distinct lipid classes, they are closely connected within the sphingolipid metabolic network. These findings support the concept that sphingolipid species with different chain lengths and saturation levels may have distinct biological roles in kidney disease.

Mechanistically, sphingolipid acyl-chain composition may affect membrane organization and cellular stress responses. Sassa et al. showed that reduction of C24 sphingolipids with compensatory accumulation of C16 sphingolipids increased cellular susceptibility to apoptosis-inducing stimuli, possibly through changes in membrane properties and lipid microdomain formation (Sassa et al., 2012). Ohno et al. further demonstrated that ELOVL1 and CERS2 cooperate in the generation of C24 sphingolipids, which have specific membrane-related properties due to their long acyl chains (Ohno et al., 2010). These findings provide a biological basis for the possibility that different sphingolipid species may exert different effects in DN.

Mechanistically, sphingomyelin may aggravate podocyte injury through membrane remodeling, ceramide-related sphingolipid signaling, and inflammatory responses. As a major membrane sphingolipid, sphingomyelin contributes to lipid raft formation and receptor-associated signaling (Simons and Toomre, 2000). Lipid rafts are important for the organization of the glomerular slit diaphragm, and podocin, a raft-associated protein, interacts with nephrin and helps maintain slit diaphragm integrity (Simons et al., 2001; Schwarz et al., 2001). Therefore, abnormal sphingomyelin accumulation may disturb membrane microdomains and slit diaphragm-associated proteins, consistent with the reduced Nephrin and Podocin expression observed in our podocyte model. In addition, sphingomyelin can be hydrolyzed to ceramide, and ceramide accumulation in podocytes has been linked to mitochondrial dysfunction, ROS production, and diabetic nephropathy progression (Woo et al., 2020). Sphingolipid signaling has also been implicated in renal inflammation and fibrosis (Huwiler and Pfeilschifter, 2018), which may partly explain the increased FN, COL1A2, and TNF-*α* expression observed after sphingomyelin treatment under high-glucose conditions. However, oxidative stress-related pathways were not directly examined in the present study and require further investigation.

Our clinical and experimental findings should therefore be interpreted as supporting sphingomyelin dysregulation in DN rather than proving that all sphingomyelin species have the same effect. In the clinical cohort, SM (d18:1/16:0) showed a progressive increase across healthy controls, diabetes-only patients, and DN patients, and total plasma sphingomyelin was elevated in DN and associated with renal function indices. In podocyte experiments, sphingomyelin treatment under high-glucose conditions aggravated podocyte injury-related markers, fibrotic/extracellular matrix-related markers, and TNF-*α* expression. Together, these findings support the involvement of sphingomyelin dysregulation in established DN and high-glucose-related podocyte injury conditions. However, the MR finding for sphingomyelin (d18:1/24:1, d18:2/24:0) should still be viewed as a genetically prioritized, species-specific signal that requires further validation.

Additionally, we identified three associated genes: *MTCO3P1, HLA-DOA,* and *BTNL2. MTCO3P1* is a pseudogene with no protein-coding function, retaining only DNA sequence characteristics. *HLA-DOA*, located in the HLA complex on chromosome 6, is an HLA class II gene. The *HLA-DOA* protein it encodes regulates the loading of antigen peptides onto HLA class II molecules, thus modulating T cell activation and adaptive immune responses. BTNL2, also located in the HLA region on chromosome 6, regulates the activation, proliferation, and differentiation of T cells, particularly γδ T cells. It helps maintain immune tolerance by inhibiting excessive immune responses. Current studies on *HLA-DOA* in kidney diseases have mainly focused on transplant rejection and immune-mediated conditions, such as membranous nephropathy, while its role in DN has not yet been investigated. These findings suggest that the SNPs identified in this study are enriched in immune function-related gene regions. Together with the increased TNF-α expression observed in sphingomyelin-treated podocytes, these genetic annotations suggest that immune and inflammatory responses may be involved in the candidate GM-sphingolipid-DN pathway. Immune regulation has received increasing attention in diabetes and its complications. Through interactions with the immune system, GM may contribute to the onset and progression of DN.

Several limitations should be noted. First, although MR can reduce confounding and reverse causation, causal inference still depends on core instrumental variable assumptions. Despite the use of F-statistic filtering, LD clumping, MR-Egger regression, MR-PRESSO, Cochran’s Q test, and leave-one-out analysis, residual horizontal pleiotropy, instrument selection bias, and other unrecognized biases cannot be fully excluded. Second, this study used summary-level GWAS data, so potential sample overlap could not be directly assessed. Given the large number of microbiota, pathway, and metabolite traits tested, nominal MR associations should be interpreted cautiously unless supported by FDR correction and consistent sensitivity analyses. In addition, the colocalization analysis used PP. H4 > 0.5 as a suggestive threshold, which is less stringent than thresholds such as PP. H4 ≥ 0.8 used in some studies; therefore, these findings should be interpreted as exploratory evidence for possible shared genetic signals rather than definitive colocalization. Third, most GWAS datasets were derived from individuals of European ancestry, whereas the clinical cohort was recruited from a Chinese population, which may limit generalizability. Fourth, although the clinical cohort included healthy controls, diabetes-only patients, and biopsy-confirmed DN patients, it was single-center, cross-sectional, and relatively small. Finally, the clinical and experimental validation mainly supports sphingomyelin dysregulation in DN, but does not directly prove the full Parasutterella-sphingomyelin-DN pathway, because stool samples were not collected. Moreover, the MR-prioritized sphingomyelin signal involved specific molecular species, whereas clinical and *in vitro* analyses included total sphingomyelin and other sphingomyelin species. Further multicenter prospective studies integrating independent metabolomic validation, metagenomic sequencing, targeted lipidomics, and animal models are needed to validate this candidate pathway. Although the podocyte experiments were expanded to include injury-related, fibrotic/extracellular matrix-related, and inflammatory-related markers, upstream sphingolipid metabolic enzymes, ceramide-related signaling, oxidative stress pathways, and other cellular stress responses were not directly examined. Further mechanistic studies are needed to clarify how sphingomyelin dysregulation contributes to podocyte injury and DN progression.

In summary, this integrative analysis provides genetic evidence supporting potential links among GM, sphingolipid-related metabolism, and DN. Clinical and experimental findings further support sphingomyelin dysregulation in DN, while the specific roles of different sphingomyelin species and the upstream microbial component require further validation. These findings provide a basis for future mechanistic studies to validate the candidate GM-sphingolipid-DN axis and to clarify how sphingomyelin dysregulation contributes to podocyte injury, extracellular matrix remodeling, inflammatory responses, and DN progression.

## Data Availability

The publicly available GWAS summary datasets analyzed in this study can be found in the IEU OpenGWAS project (https://opengwas.io/datasets) under accession numbers ieu-a-1100, ieu-a-1103, and finn-b-DM_NEPHROPATHY, and the GWAS Catalog (https://www.ebi.ac.uk/gwas) under accession numbers GCST90199621–GCST90201020.
