# Supplementary figures and images for: Genetic and clinical evidence implicates a potential Parasutterella–sphingomyelin pathway in diabetic nephropathy
Source: Front Microbiol. 2026 Jul 16;17:1848678. doi: 10.3389/fmicb.2026.1848678 (PMC13422506; doi:10.3389/fmicb.2026.1848678)

Fig 8. E

|          |   |   |   |   |     |                      |
|----------|---|---|---|---|-----|----------------------|
| HG       | - | - | + | + | +   | +                    |
| Mannitol | - | + | - | - | -   | -                    |
| Eth      | - | - | - | + | +   | +                    |
| SM       | - | - | - | - | 200 | 250                  |
|          |   |   |   |   |     |                      |
|          |   |   |   |   |     | ( $\mu\text{g/mL}$ ) |
|          |   |   |   |   |     | KDa                  |

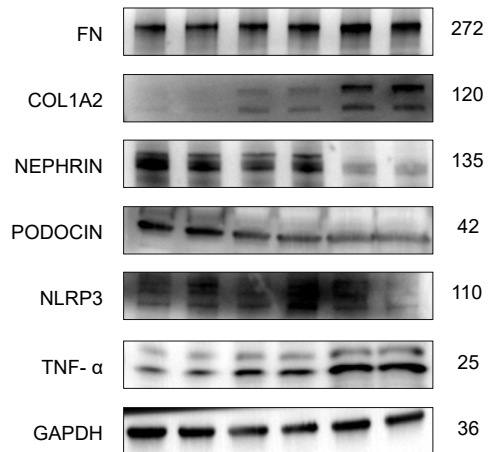

180

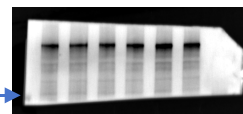

130

100

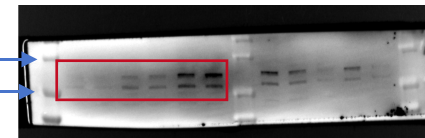

180

130

100

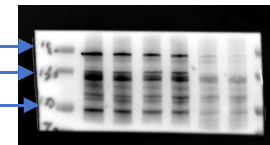

55

40

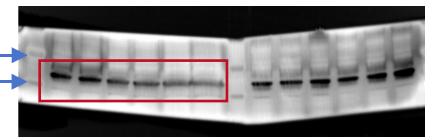

180

130

100

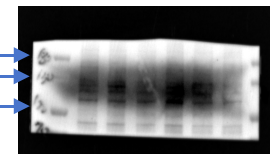

35

25

15

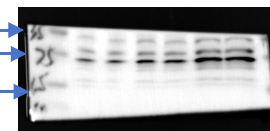

40

35

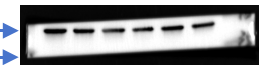

Supplement: Supplementary file 1 [file Data_Sheet_1.pdf]
