# Supplementary material for: Genetic and clinical evidence implicates a potential Parasutterella–sphingomyelin pathway in diabetic nephropathy
Source: Front Microbiol. 2026 Jul 16;17:1848678. doi: 10.3389/fmicb.2026.1848678 (PMC13422506; doi:10.3389/fmicb.2026.1848678)
Supplement: Supplementary file 2 [file Supplementary_file_1.docx]

**Supplement Methods**

**Patients and specimen preparation**

Patients were recruited at Zhengzhou university people’s hospital (Zhengzhou, China). This study was performed following the ethical guidelines of the declaration of *Helsinki* and was approved by the local ethical committee (Ethics Approval No. 2020207). Written informed consent was obtained from all participants before collection of specimens and clinical information. Healthy controls from the health management center, and patients with DN from the department of nephrology. All participants underwent venipuncture of the median cubital vein by a trained healthcare professional, with 5 ml of fasting blood collected after at least 8 hours of overnight fasting. After collection, the tubes containing ethylene diamine tetraacetic acid were gently shaken several times, then centrifuged at 3000 r/min and 4°C for 10 minutes. The supernatant plasma was collected and divided equally among several cryopreservation tubes. After 15 minutes rapid freezing in liquid nitrogen, all plasma samples were stored at -80°C for future use.

Healthy controls, patients with diabetes mellitus without diabetic nephropathy, and patients with biopsy-confirmed DN were recruited from a Chinese clinical cohort. The diabetes-only group was included as a disease-control group to help distinguish diabetes-related metabolic changes from DN-related changes. After an overnight fast of at least 8 hours, 5 mL of venous blood was collected from the median cubital vein by trained personnel. Inclusion criteria for the DN group were age 18-75 years and biopsy‑confirmed DN. Exclusion criteria were: (1) renal biopsy showing other renal pathologies in addition to DN. (2) end‑stage renal disease requiring peritoneal dialysis or hemodialysis. Subjects were included if they met the diagnostic criteria for DM established by the American Diabetes Association criteria defined as fasting plasma glucose ≥ 7.0 mmol/l, 2-hour plasma glucose ≥ 11.1 mmol/l during an oral glucose tolerance test, or random plasma glucose ≥ 11.1 mmol/l with typical hyperglycemic symptoms; aged between 18 and 75 years old; with glycated hemoglobin levels ranging from 6.5% to 12%. Exclusion criteria were gestational diabetes or other special types of diabetes. Healthy controls were individuals who underwent routine health examinations at our hospital’s health management center during the same period and had normal results. Written informed consent was obtained from all participants. Plasma lipidomics was performed using LC-ESI-MS/MS (QTRAP® 6500+; Sciex). The detailed parameters and quality control protocols for LC-MS/MS are provided in the supplementary methods. Baseline characteristics and candidate metabolites were compared among the three groups. Continuous variables were presented as median (interquartile range) and compared using the Kruskal-Wallis test. Categorical variables were presented as n/N (%) and compared using the chi-square test or Fisher’s exact test, as appropriate. Pairwise comparisons were performed with multiple-testing correction where applicable, and adjusted P values were reported for post hoc group comparisons.

**Detection of blood metabolites**

***Sample preparation and extraction***

The plasma samples were removed from the -80 °C refrigerator and thawed on ice until no ice crystals remained. Post-thaw, samples were vortexed for 10 s to ensure thorough mixing, and 50 ul of each sample was transferred to a labeled centrifuge tube. A total of 1 ml lipid extraction solution (MTBE: MeOH =3:1, v/v) containing internal standards was added, followed by vortexing for 15 min. Subsequently, 200 ul of water was added, and the mixture was vortexed for 1 min, then centrifuged at 12,000 r/min for 10 min at 4 °C. After centrifugation, 200 μl of the upper organic layer was collected and evaporated using a vacuum concentrator. The residue was reconstituted with 200 ul lipid reconstitution solution (acetonitrile:isopropanol = 1:1, V/V), vortexed for 3 min, and centrifuged at 12,000 r/min for 3 min. The dry extract was dissolved in 200 μl reconstituted solution (ACN: IPA=1:1, v/v) to LC-MS/MS analysis.

***HPLC Conditions***

The sample extracts were analyzed using an LC-ESI-MS/MS system (UPLC, ExionLC AD，https: //sciex.com.cn/; MS, QTRAP® 6500+ System, https://sciex.com/). The analytical conditions were as follows, UPLC: column, Thermo Accucore™C30 (2.6 μm, 2.1 mm×100 mm i.d.); solvent system, A: ace tonitrile/water (60/40,V/V, 0.1% formic acid, 10 mmol/l ammonium formate), B: acetonitrile/isopropanol (10/90 VV/V, 0.1% formic acid, 10 mmol/l ammonium formate); gradient program, A/B (80:20, V/V) at 0 min, 70:30 V/V at 2.0 min, 40:60 V/V at 4 min, 15:85 V/V at 9 min, 10:90 V/V at 14 min, 5:95 V/V at 15.5 min, 5:95 V/V at 17.3 min, 80:20 V/V at 17.3 min, 80:20 V/V at 20 min; flow rate, 0.35 ml/min; temperature, 45 °C; injection volume: 2 μl. The effluent was alternatively connected to an ESI-triple quadrupole-linear ion trap (QTRAP)-MS.

***ESI-MS/MS Conditions***

Linear ion trap and triple quadrupole (QQQ) scans were acquired using a triple quadrupole-linear ion trap mass spectrometer (QTRAP® 6500+ LC-MS/MS System; Sciex, Framingham, MA, USA) equipped with an electrospray ionization (ESI) Turbo Ion-Spray interface. The instrument operated in both positive and negative ion modes and was controlled by Analyst 1.6.3 software (Sciex). The operating parameters of the ESI source were set as follows: ion source type, Turbo Spray; source temperature, 500 °C; ion spray voltage (IS), 5500 V (positive ion mode) and -4500 V (negative ion mode); ion source gas 1, gas 2, and curtain gas were set to 45, 55, and 35 psi, respectively. Instrument tuning and mass calibration were performed using 10 μmol/l and 100 μmol/l polypropylene glycol solutions for the QQQ and LIT modes, respectively. QQQ scans were acquired in multiple reaction monitoring mode, with collision gas (nitrogen) set to 5 psi. Declustering potential and collision energy for each individual MRM transition were optimized further to ensure signal sensitivity. A specific set of MRM transitions was monitored for each time period, corresponding to the metabolites eluted during that period.

***Data analysis process***

Data were analyzed sequentially. orthogonal Partial least squares–discriminant analysis (OPLS-DA) was first performed to visualize global differences among study groups and to evaluate lipid species contributing to group discrimination, with variable importance in projection (VIP) scores calculated accordingly. Subsequently, pairwise comparisons of lipid abundances between groups were conducted using Student’s t-test for normally distributed data or the Wilcoxon rank-sum test for non-normally distributed data. To control for inflated false-positive rates due to multiple testing, raw p-values were adjusted using the Benjamini–Hochberg (BH) method to control the false discovery rate (FDR). Differentially abundant lipids were defined as those with FDR-adjusted q-values < 0.05. VIP scores (>1) derived from PLS-DA were used to indicate lipid species with strong contributions to group separation, but were not used as criteria for statistical significance. Kyoto Encyclopedia of Genes and Genomes (KEGG) pathway enrichment analysis was subsequently performed on DN-specific differentially abundant lipids using the hypergeometric test. Enrichment p-values were corrected for multiple testing using the BH method, and pathways with adjusted q-values < 0.05 were considered statistically significant.

**Western blotting (WB)**

Kidney tissues and cells were resuspended in RIPA buffer containing 1x protease inhibitor cocktail and 1x phosphatase inhibitor. Tissues were homogenized to lysate in 1.5 ml lysing matrix tubes using tissue homogenizer. Tissue lysates and cells lysates were incubated in ice for 20~30 minutes to ensure proper cell lysis, then centrifugation of lysates at 4 °C for 15 min at 12,000 r/min. The lysates were heated (99 °C) for 10 min with SDS-PAGE loading buffer. Proteins were then separated by polyacrylamide gel electrophoresis in acrylamide gels (4~20%) and transferred onto PVDF membranes, which were immediately placed in 5% non-fat milk in TBS-Tween (TBST) buffer for blocking (1~1.5h at room temperature). Membranes were then washed in TBST buffer for 15 min, followed by incubation with specific primary antibodies at 4 °C overnight. Membranes were then washed 3 times for 30 min in TBST buffer, and incubated with specific secondary antibodies at room temperature for 1 h.

**Measurement of SM**

Quantitative measurement of SM was performed in 10 µl of plasma from health subjects and patients with DN, DM using a human SM colorimetric Assay Kit (cat.no. 10009928, Cayman). 1 ml SM enzyme mixture, 0.5 ml sphingomyelinase, 10 µl SM alkaline phosphatase, and 490 µl diluted buffer were added to the SM color detector to make a 5 ml reaction mixture, and the mixture was stable for 24 h at 4°C. 10 µl standard (tubes A–G) was added to designated wells, followed by 10 µl undiluted plasma to three wells per sample. Reactions were initiated by adding 100 µl reaction mixture to each well; the plate was gently shaken for a few seconds, covered, and incubated on a shaker at room temperature for 60 min. Absorbance was read at 585-600 nm using a plate reader.

**Statistical analysis**

Statistical analyses were conducted using R software (Version 4.5.1; https://www.R-project.org). Continuous and categorical variables were analyzed using appropriate statistical tests according to their distribution and measurement level. For continuous variables, normality was assessed using the Shapiro-Wilk test and homogeneity of variance using Levene’s test. Variables meeting both assumptions were compared using one-way analysis of variance, with Tukey’s post hoc test for pairwise comparisons. If normality or homoscedasticity assumptions were violated, the Kruskal-Wallis test was used. Continuous variables are presented as mean ± standard deviation (SD) or median (interquartile range, IQR), as appropriate. For categorical variables, group differences were assessed using the chi-square (χ2) test. Fisher’s exact test was applied when any expected cell count was less than 5. For variables with multiple categories or sparse data, Fisher’s exact test with simulated p-values was used. Categorical variables are reported as counts and percentages. Correlation analyses were performed using *Pearson’s* correlation test for normally distributed data or *Spearman’s* rank correlation test for non-normally distributed data. All tests were two-sided, and P<0.05 was considered statistically significant.

**Supplement Figures**


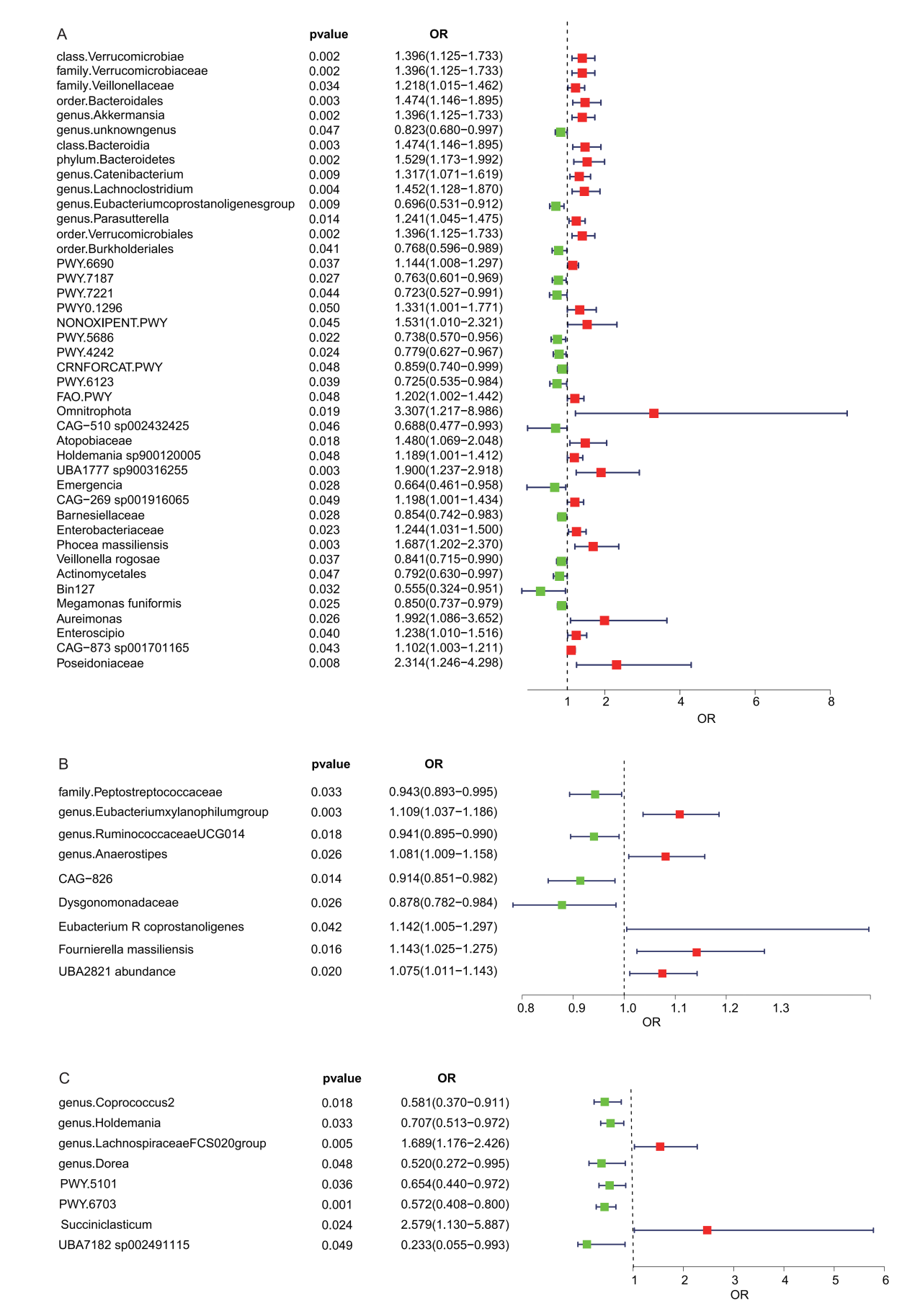


**Supplementary Fig. 1** Forest plot of Mendelian randomization analysis for the causal relationship between gut microbiota and diabetic nephropathy. (A) GM to DN. (B) GM to eGFR. (C) GM to UACR.


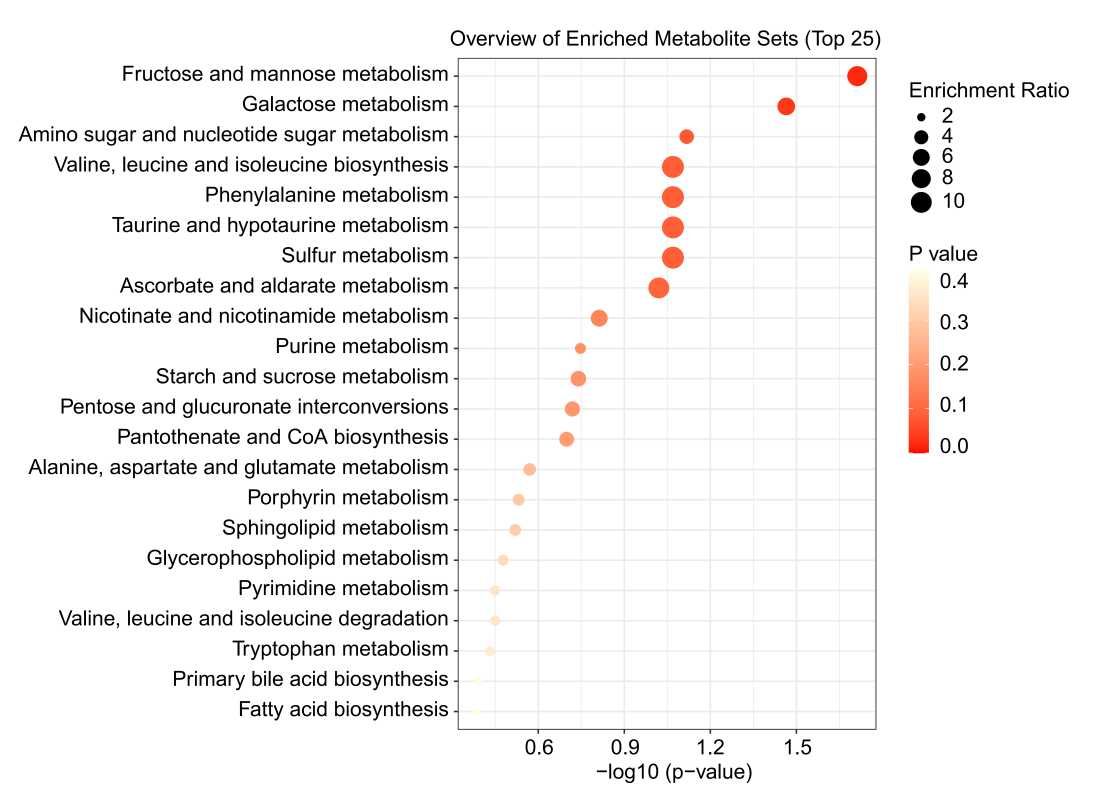


**Supplementary Fig. 2** Bubble plot showing the results of the enrichment analysis for metabolites related to the DN phenotype


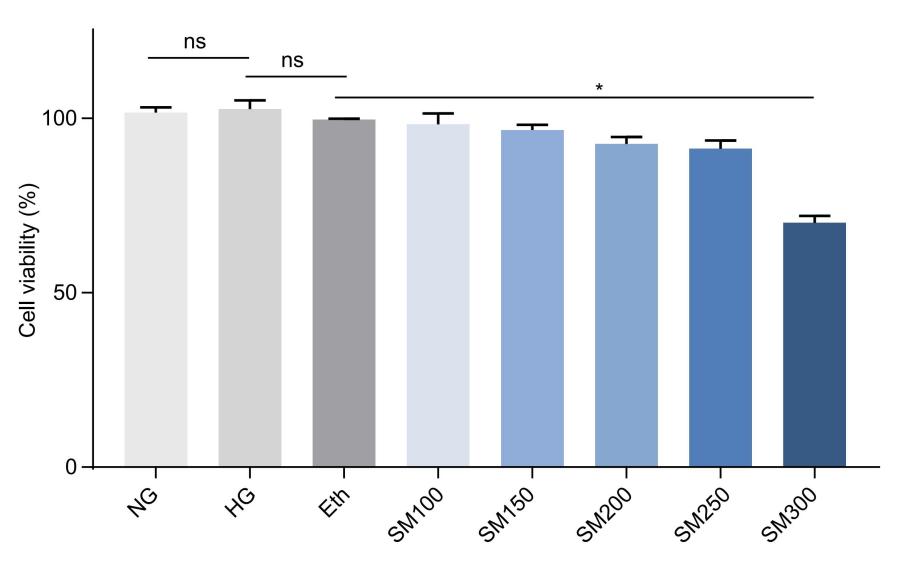


**Supplementary Fig. 3** CCK-8 assay for selecting sphingomyelin concentrations in podocytes
